# Tutors and self-assessment of medical students’ performance in pre-clinical problem-based learning tutorial sessions

**DOI:** 10.15694/mep.2018.0000265.1

**Published:** 2018-11-20

**Authors:** Mary Anne Cordero, Najwa Abdur Rashid, Raja El Hasnaoui, Kavitha Ganesh, Zenat Khired

**Affiliations:** 1 Princess Nourah Bint Abdulrahman University

**Keywords:** Problem-based learning, self-assessment, tutors assessment, self-learning, medical students, PBL hybrid curriculum

## Abstract

This article was migrated. The article was marked as recommended.

**Background:** It is currently acknowledged that developing the skills in self-assessment is imperative for the continuing learning of every physician.

**Objective** **:** This study aimed to assess students’ performance in tutorial sessions in a hybrid problem-based learning (PBL) curriculum conducted during the pre-clinical years through self-and tutors assessment.

**Methods** **:** Sixty nine first year and eighty second year students of the Bachelor of Medicine and Bachelor of Surgery (MBBS) Program at the Princess NourahBint Abdulrahman  University  participated in the study. Both self-and tutors assessment of students performance within the PBL tutorial setting was conducted using a validated questionnaire developed by Valle et al., (1999). Mean ratings between self-and tutors assessments were compared using paired *t-*test. Association between self and tutors  assessments  and PBL written exam scores was analyzed using Pearson correlation coefficients.

**Results:** Results revealed a significant difference between self and tutors assessment for first year medical students under scoring their own performance than the tutors’ with a total mean score of  79.99 (± 25.87) and 98.02 (± 8.71) respectively ( *P*=0.001).Tutors assessment correlated poorly with self-assessment (r=0.344). No correlation between self-assessment and exam scores was observed but a strong correlation  (r=0.726 & *P*=0.045) was shown for tutor assessment and exam scores. In contrast to the first year group, the total score showed no significant difference between self- and tutors assessment in second year students. There was strong correlation between tutor and self-assessment (r=0.722& *P*=0.041) as well as between tutor assessment and exam scores (r=0.806 & *P*=0.030 ) but not with self-assessment and exam scores.

**Conclusion:** This study has presented reservations with regards to the use of self-assessment scores as part of the overall student grade in PBL.

## Introduction

Problem-based learning (PBL) is a method of small group collaborative learning that was first used in Medical Education at McMaster University in the late 1960s and currently being used in a number of medical schools worldwide. This approach to learning has been advocated as a way to provide a better learning environment for students in healthcare professions ( Hmelo-Silver & Barrows, 2006; Susarla et al., 2004). Various skills like communication, collaboration, critical thinking and problem solving can be stimulated and developed by PBL ( Hmelo-Silver, 2004; Savery, 2006).

In PBL, students are required to participate actively in their own learning by researching and working through a series of real-life problems which are used as a motivational context to drive their learning process ( Arambula-Greenfield, 1996). During the PBL tutorial sessions, the students use the content from lectures and independent study to approach cases under the guidance of the facilitator ( Epstein, 2004). Students learn group-work skills and attitudes and improve their communication skills in PBL ( Wood, 2003). These skills and attitudes include teamwork, cooperation, respect for colleagues’ views, chairing a group, and interaction with group members ( Wood, 2003). It is therefore believed that PBL is good tool for group learning ( Dolmans et al., 2005).

A number of studies support the benefits of PBL as an innovative approach in health professions education like the structuring of knowledge for its use in clinical contexts. Also included are the development of an effective reasoning process, the development of effective self-directed learning skills, as well as stimulating students’ motivation for learning (( Morales-Mann &Kaitell, 2001; Fincham & Sculer, 2001). The use of PBL and its positive outcomes within the medical student curriculum has also been widely studied Taylor & Miflin, 2008). It was reported that students who graduated from a PBL curriculum may have less confidence in their knowledge but better clinical performance than those from traditional medical schools ( Watmough et al., 2010). They also demonstrate an ability to work more efficiently ( Schmidt et al., 2006b). Moreover, students in PBL programmes show a greater tendency to use evidence-based medicine ( Thomas, 1997). In terms of cognitive domain, Dolmans & Schmidt (1996) found that there are several cognitive effects of PBL on student learning like an increased retention of knowledge, enhancement of integration of basic science concepts into clinical problems, development of self-directed learning skills, and the enhancement of students’ intrinsic interest in the subject matter. Prince et al., (2005) also stated that  PBL medical graduates more likely learned communications skills and teamwork than the non- PBL graduates.

Students’ self-assessment in PBL is encouraged as part of their learning and critical assessment process. It supports learners in discovering their strengths and weaknesses in learning ( Leach, 2012). Self-assessment is considered as an important part of learning process where students apply self-regulatory processes in their learning. They set their own goals in learning, select learning strategies, assess learning progress, evaluate information from feedback, and then improve their learning processes for the next PBL tutorial session ( Loyens et al., 2008). Also, in self-assessment, students compare their own performance to specific standards or to others’ performance, thus stimulating them to learn. It is believed that students who maintain a more active role in the learning process are able to assess better their own performance ( Eva, 2001).

Since its commencement in the year 2012, the College of Medicine of Princess Nourah Bint Abdulrahman University (PNU), Kingdom of Saudi Arabia, has adopted a unique Problem-based hybrid curriculum. The first two years of the five-year Bachelor of Medicine and Bachelor of Surgery (MBBS) Program focuses on the basic medical sciences which is delivered in a block system. The implementation of the hybrid PBL curriculum at PNU also demands for the necessity to evaluate the processes and outcomes of this curricular innovation. Since the beginning of the implementation of hybrid PBL curriculum, no tutorial-based assessment of students’ progress during PBL tutorial session has been conducted. The Faculty of Medicine has felt the need to evaluate whether or not the objectives and fundamental components of PBL method are achieved. There are four factors which reflect the essential components of problem-based learning: independent study, group interaction, reasoning skills, and active participation ( Barrows,1985; Walton & Matthews,1989).

This study explored students performance in PBL tutorial sessions based on tutor’s assessment and based on how students assess their own performance. Comparison between self- and tutors evaluations as well as the potential association between the tutor and self-evaluation with the PBL written test score was investigated. The PBL assessment questionnaire used in this study was developed by a team of physicians trained in PBL educational strategies ( Valle et al., 1999). The assessment tool includes the 4 fundamental components of PBL namely; independent study, group interaction, reasoning abilities, and active participation.

Findings of this study may provide useful insights that may help students identify areas in which they need to further enhance their learning thus; they will be encouraged to take responsibility for their own learning process.  Data from both self and tutors assessments may also be beneficial for the PBL tutors to identify areas in the delivery of PBL tutorials in which students require more guidance. It may also provide feedback to recognize which components of the curriculum needs improvement especially on the formative and summative assessments used to evaluate the first year and second year medical students.

## Methods

### Subjects

The subjects of the study  were the fourteen PBL tutors,  sixty nine first year and eighty second year students enrolled in Bachelor of Medicine and Bachelor of Surgery (MBBS) Program at the College of Medicine of Princess NourahBint Abdulrahman University.  The first year students  had completed  at least 25  PBL tutorial sessions while the second year had completed at least 40 tutorial sessions and both were evaluated at the end of the year Block.

### Setting

The MBBS Program at Princess Nourah Bint Abdulrahman University starts with a 2-year preclinical basic sciences program which is delivered through blocks system. These include the Foundation Block, Musculoskeletal, Cardiovascular, Respiratory, and Renal Blocks for first years students. Central Nervous System, Endocrine, Gastrointestinal and Hematology, and Reproduction  Blocks are for the second year students.  In each block, basic science subjects like Anatomy, Physiology, Biochemistry, Genetics, Pathology, Microbiology, and Pharmacology are integrated according to the themes of the weeks. Lectures, practical, learning skills, medical professionalism, self-directed learning (SDL), and clinical skills sessions as well as  problem based learning (PBL) are given within each block. The PBL in every block is delivered in two tutorial sessions which are usually scheduled for 2 hours per session and facilitated by a tutor. During first tutorial session, a group of 10 to 12 students analyze, try to explain the problem, and formulate learning objectives. Through self-directed study, students acquire additional information from different references. After 3 to 4 days, the second session is given and the same group of students put together their new acquired knowledge and further explain and answer the questions asked in the problem. The students analyze their learning processes, newly acquired knowledge and group performance.

### Data Collection and Analysis

Data were collected by giving out validated assessment questionnaire which was an  adaptation of a 24-item rating scale questionnaire developed by Valle et al., (1999). The questionnaires were hand distributed to the students and tutors at the end of the academic year.

The total scores of students’ performance based on self and tutors evaluation were tallied and entered into EXCEL and then imported into the Statistical Packages for Social Sciences (SPSS Version 23) for analysis. Comparisons of the mean ratings between self- and tutors evaluations were done using paired t-test. Association between self- and tutors  assessments  and PBL written exam scores was analyzed using Pearson correlation coefficients.

### Instrument

The performance of the students in PBL tutorial sessions was evaluated using a validated questionnaire adapted from Valle et al., (1999).  The assessment questionnaire was developed  by a team of medical teachers, psychologists, and education specialists from the Faculty Medicine at the National Autonomous University of Mexico (UNAM) who were trained in PBL educational strategies. The instrument was developed to enable PBL tutors to assess students’ attitudes and competencies acquired through their participation in PBL tutorial sessions. The assessment questionnaire may be used to track the evolution of attitudes and skills during PBL tutorials, to serve as a tool for providing useful feedback for students, and to evaluate the overall tutorial group performance at the end of the course ( Valle et al., 1999).

The questionnaire includes 24 items clustered into 4 factors which are important components of  PBL. The first factor, independent study, comprises 9 items linked to students’ initiative, motivation, and persistence in searching for information, studying, and achieving the learning objectives and tasks agreed on by the group. The second factor which refers to group interaction, includes 5 items which concern students’ ability to function in a group, such as openness to suggestions and decisions adjusted to different group roles and respect towards their peers. The third factor, reasoning ability, includes 6 items related to students’ ability to analyze cases, formulate hypothesis, and clarify concepts. The fourth factor, active participation, includes 4 items reflecting a specific form of interacting with the group that shows behavior such as contributing, helping and sharing reflections, ideas and knowledge.

Each item in the questionnaire was rated on a 5-point Likert scale from 1(never) to 5 (always). Scores on all four factors (PBL components) of the self- and tutors assessments were summed to give an overall score. A pilot study on 20 students was conducted to confirm the applicability of the questionnaire. The 4 factors had a Cronbach reliability coefficients of 0.95, 0.83, 0.94, and 0.93 respectively with a total reliability of 0.96 ( Valle et al., 1999).

## Results/Analysis

### Students performance in PBL tutorial sessions based on tutors and self-evaluation

Levels of agreement between scores for self-assessment and tutors assessment were explored. Among the 72 first year medical students enrolled, 69 were evaluated using the Valle et al., (1999) PBL assessment questionnaire. Three (3) students dropped the course in the middle of the year. Table 1 shows the differences between self-and tutors evaluationsof first year student’s performances in the PBL tutorial sessions. Paired *t-*test revealed students under marking their own performance in the four fundamental components of PBL: independent study, group interaction, active participation, and reasoning abilities. The first year studentsscored themselves significantly lower than their tutorswith a total mean score of 79.99 (± 25.87) and 98.02 (±8.71) respectively with *P*=0.001.

**Table 1.** Self and tutors assessment for first year medical students (N= 69)

PBL components	Self-assessmentMean ± SD	Tutors assessmentMean ± SD	*t*	*P*
Independent Study	28.65 ± 8.29	37.34 ± 3.66	-8.28	0.001
Group Interaction	17.41 ± 6.64	21.71 ± 2.82	-5.38	0.001
Active Participation	13.65 ± 5.17	16.98 ± 2.57	-5.30	0.001
Reasoning Abilities	20.27 ± 7.51	23.03 ± 2.57	-3.12	0.003
Total Score	79.99 ± 25.87	98.02 ± 8.71	-6.07	0.001

*P= < 0.05*

Eighty two (82) second year students were enrolled and participated in the study but 2 of them did not complete the self-assessment questionnaire. Thus only the assessmentsof 80 students were considered in data analysis. The comparisons of self- and tutors evaluations for second year medical students are shown in Table 2. Statistically significant difference between self- and tutors assessment was present only forindependent study ( *P*= .004) with means and standard deviations of  30.38 ± 10.55 and  34.70±6.22 respectively. In contrast to the first year group, there was no significant difference between self- and tutors assessment in group interaction, active participation, and reasoning abilities among the second year students.

**Table 2.** Self- and tutors assessmentfor second year medical students (N=80)

PBL components	Self-assessmentMean ± SD	Tutors assessmentMean ± SD	*t*	*P*
Independent Study	30.38 ± 10.55	34.70 ± 6.22	-3.01	0.004
Group Interaction	18.35 ± 7.05	19.82 ± 3.52	-1.53	0.134
Active Participation	14.84 ± 5.61	14.10 ± 2.95	0.97	0.336
Reasoning Abilities	21.43 ± 7.46	22.06 ± 4.64	-0.62	0.537
Total Score	85.0 ± 29.27	90.68 ± 15.03	-1.46	0.150

*P= < 0.05*

### Relationship between students PBL scores in written exam and self/tutors evaluations

Table 3 displays the descriptive statistics of students’ written exam scores given at the end of each block.  Correlation between self- and tutors’ assessment and exam score was explored andthe results are shown in Table 4. We used Pearson correlation coefficient to test for the strength and direction of linear relationships between self- and tutors assessments with student scores in PBL written exam.

For first year students, tutors assessment correlated poorly with self-assessmentas shown in Table 4(r=0.344). No correlation was observed between self-assessment and exam scores but a strong correlation (r=0.726 & *P*=0.045) was shown for tutors assessment and exam scores as shown in Table 3. In contrast with the first year students, strong correlation was observed between tutors assessmentand self-assessment (r=0.722 & *P*=0.041) among second year students. Likewise, a strong correlation (r=0.806 & *P*=0.030) was observed between tutors assessment and exam scores but not with self-assessment and exam scores.

**Table 3.** Students scores in PBL written examinations

Written test	N	Mean	Standard Deviation	Minimum Score	Maximum Score
**First Year**	69	87.62	7.89	60.67	97.12
**Second Year**	80	88.31	7.47	60.12	98.00

*(written exam scores are expressed in percentage)*

**Table 4.** Correlation between self-and tutors assessment and scores in written test

Variables	Correlation (r)	P-value
**First Year**		
Self- Assessment –Tutors’ Assessment	0.344	0.107
Self- Assessment – Written Test	-0.063	580
Tutor’s Assessment – Written Test	0.726	0.045
**Second Year**		
Self- Assessment –Tutors’ Assessment	0.722	0.041
Self- Assessment – Written Test	0.189	0.160
Tutor’s Assessment – Written Test	0.806	0.030

*P= <0.05*

## Discussion

PBL highlights the development of self-learning where students take the responsibility of assessing their own cognition in the process of learning. Presently, it is acknowledged that developing the skills in self-assessment is essential for the continuing learning of every medical doctor. Medical students are expected to be cognizant of their own learning potentials as well as their difficulties in order to improve their performance. Self-assessment of one’s performance is encouraged among students as part of their learning and critical assessment process. It supports learners in discovering their own strengths and weaknesses ( Leach, 2012) and it is considered as an important part of learning process where students apply self-regulatory processes in their learning. Students set their own goals, select learning strategies, assess their progress, evaluate information from feedback, and then improve their learning processes for the next tutorial ( Loyens et al., 2008).

Results of our study showed that the first year medical students consistently underscored their own performance in PBL tutorial sessions. Tutors gave significantly higher scores in all factors; independent study, group interaction, active participation, and reasoning ability. These findings confirm what have been reported in previous studies. Indeed, self-assessment of tutorial processes in PBL is not an exact measure because students under-marked their own performance ( Das et al., 1998; Sullivan et al., 1999; Reiter et al., 2002). In addition, it was also reported that self-assessment has low accuracy in some students. Students admitted to MBBS program having degrees in arts, commerce, music, education or law have significantly higher initial self-assessment scores ( Papinczak, 2007). Nevertheless, generally, students score themselves generously above their tutors  marks ( Machado, 2008).

Compared with the first year students, the second year students were able to assess their performance in concordance with tutor’s assessment which is considered to be the gold standard in assessment. Although tutors’ assessment mean scores were generally higher in 4 factors, it was not statistically significant except for independent study. This finding is similar with the previous studies conducted in which self-evaluation scores were observed to be higher as students progressed over a period of time. It was reported that students’ initial self-assessment significantly increased as they gained more experience ( Woolliscroft et al., 1993; Rezler, 1989). In this study, the second year students are already acquainted with the PBL tutorial sessions since they were in first year and have taken at least 40 tutorial sessions at the culmination of the Reproduction Block when assessments were conducted.

Worth noting was the poor correlation between self-assessment and tutors assessment among first year compared with a strong correlation among second year students. As presented in the preceding paragraphs, possible reason for this was the tendency of the first year students to underscore their own performance. Our freshmen students were accustomed to lecture method since their high school and Preparatory Year College before embarking into Hybrid PBL curriculum in the College of Medicine.  We could hypothesize that the lack of experience in PBL for students might have affected their evaluation of their own performance. Being new to the PBL method, they may not have yet fully acquired the experience and develop the confidence to assess their own learning performance.  Unlike the first year, second year students had already almost two years of experience to PBL curriculum and thus they are already familiar with the entire PBL tutorial process.

A strong correlation of tutor’s assessment with PBL written exam scoreswas observed but in contrary, poor correlation of self-assessment with the written exam scores was revealed in both first year and second year medical students. Again, this may imply inaccuracy of self-assessment because students may either underscore or over score their own performance. As pointed out by Mattheos et al., (2004), students have the tendency to overestimate their competence especially the low performing ones ( Edwards et al., 2003). As reported by Eva et al., (2004), low correlation between students’ self-assessment and performance on a test of medical knowledge was observed in a sample of medical students and there was no evidence of improvement even after finishing one year of medical education.

Part of the summative assessment that we currently practice in PNU, Collegeof Medicine is the tutors’ assessment and the PBL written examination given at the end of each block. For written PBL exam, students are given two cases to analyze then to answer the questions after each case. Written PBL exam comprises 10%, while tutors’ assessment covers 5% of the total summative assessment mark for each block.We do not consider self-assessment as either formative or summative assessment yet we acknowledged the importance of self-assessment as formative method of assessment.  In general, results of this study revealed inaccuracy of self-assessment in first year and no correlation between self-assessment and written test scores for both second year and first year students. With these findings, it is worth considering inclusion of a comprehensive orientation program on the PBL objectives vis-à-vis the importance of self-assessment for first year students during the first block (Foundation Block). This may help augment students’ skills on how to evaluate their own performance appropriately. Despite reports of no significant relationship between self-assessment scores and examination results ( Tousignant & Des Marchais, 2002; Eva et al., 2004) and poor correlation between self- and tutors evaluations within medical PBL programs ( Sullivan et al., 1999; Reiter et al., 2002), it is still acknowledged that self-assessment has an important role to play in supporting the development of skills in reflection and self-awareness ( Papinczak, 2007). Self-assessment is a way of identifying one’s strengths and weaknesses to guide goal setting and enhance self-efficacy ( Eva and Regehr, 2005). It should be noted however, that as self-assessment skills improved,  cautions against over-confidence must be taken into consideration because improvement also require training, clear learning goals and the provision of feedback ( Gordon, (1991).

## Conclusion

The second year medical students in this study were able to assess their own performance in accordance with the tutors’ assessment but this was not revealed among the first year students. For both groups, self-assessment scores were not correlated with the PBL written exam scores but correlation was observed between tutors assessment and exam scores. This study has mounted reservations with regards to the use of self-assessment scores as part of the overall student grade during PBL tutorials. Further study may help us comprehend better why students are unable to assess their own performance accurately. A qualitative research may be conducted to understand how experiences and exposures to PBL tutorials enhance students’ self-assessment skill.

## Take Home Messages

• Second year medical students are able to assess their own performance in accordance with the tutors’ assessment.
• First year medical students significantly under scored their own performance compared with the tutors’ assessment.
• Self-assessment scores are not correlated with the PBL written exam scores but the tutors’ assessment scores correlated with exam scores.
• Self-assessment scores reflect inaccuracy to be considered as part of the overall students’ grade in PBL.

## Notes On Contributors

**Mary Anne W. Cordero** is a Biology Professor of the Department of  Basic Sciences  in the College of Medicine at Princess Nourah Bint Abdulrahman UniversityRiyadh, Kingdom of Saudi Arabia

**Najwa Abdur Rashid** is an Assistant Professor of Physiologyin the Department of  Basic Sciences in the College of Medicine at Princess Nourah Bint Abdulrahman UniversityRiyadh, Kingdom of Saudi Arabia.

**Kavitha Ganesh** is a Professor of Anatomyin the Department of  Basic Sciences in the College of Medicine at Princess Nourah Bint Abdulrahman UniversityRiyadh, Kingdom of Saudi Arabia **.**

**Raja El Hasnaoui** is an Associate Professor of Physiologyin the Department of Basic Sciences  in the College of Medicine at Princess Nourah Bint Abdulrahman UniversityRiyadh, Kingdom of Saudi Arabia.

**Zenat Khired** is the Head of the Department of Basic Sciences in the College of Medicine, Princess Nourah Bint Abdulrahman University and Consultant of orthopedic hip and knee arthroplasty in King Abdul Aziz University Hospital.
