# Supplementary material for: Tutors and self-assessment of medical students’ performance in pre-clinical problem-based learning tutorial sessions
Source: MedEdPublish (2016). 2018 Nov 20;7:265. [Version 1] doi: 10.15694/mep.2018.0000265.1 (PMC10711975; doi:10.15694/mep.2018.0000265.1)
Supplement: Peer review history

# Reviewer report: Reviewer response for version 1

**DOI:** 10.21956/mep.19709.r29484

**Published:** 2019-05-11

**Authors:** Po-Wei Chen

**Affiliations:** 1 Tzu Chi University, School of medicine, Department of medicine

This review has been migrated. The reviewer awarded 4 stars out of 5 >>StrengthIt is an interesting article about the ability of self-assessment for first- and second-year students in the context of the problem-based hybrid curriculum. The author also reviewed several previous studies regarding self-assessment which can be a useful reference for teachers in this field. >>SuggestionTo strengthen the article, I would try to give some ideas from a student’s view.1. In the correlation part, authors comparing the relationship between self- and tutor-assessment with the written test score. It will increase validity by knowing how the written test score is assessed. Is it in short-answer questions or choice questions? And who is in charge of marking the examination? If it’s the tutor than there may be some bias for them to be self-consistency. 2. You mentioned that you adapted the questionnaire from Valle et al. (1999). I guess it means you translate it to the Arabi. For a translated questionnaire, there should be experts to translate the questionnaire from the original language to Arabi and back to the original language to check the consistency with the initial questionnaire. Besides, researchers should do either exploratory or confirmatory factors analysis for validation.On the other hand, it will be great to know what the results of your new program to enhance first-year students’ self-assessment which can compare with this study results.>>For whom to read1. Teachers who also in the context of the hybrid problem-based curriculum.2. Teachers who are interesting in improving student’s ability of self-assessment.

Reviewer Expertise:

NA

No decision status is available

# Reviewer report: Reviewer response for version 1

**DOI:** 10.21956/mep.19709.r29485

**Published:** 2018-11-27

**Authors:** Sandra Kemp

**Affiliations:** 1 Curtin Medical School, Curtin University

This review has been migrated. The reviewer awarded 3 stars out of 5 The topic of student self-assessment is of interest and has been the subject of much empirical work, particularly in the broader educational literature. I would recommend reference to this work, as part of the introduction and discussion.A small point to note is that instruments are not inherently valid. Use of the phrase "validated assessment questionnaire" should be reworked. Evidence for validity is always context specific so it would be useful to know what processes were used for piloting and adaptation of the instrument. Data related to the factor loadings should be presented and discussed. It is not clear that the factors found in the original work were replicated and that is important for readers to know.It would be helpful for the authors to reflect on and discuss the limitations of the study in the Discussion section. This would strengthen the paper.I would encourage the authors to consider ways in which self-assessment can be used for learning purposes. Were students trained in how to self assess? Could the data be used for reflection or other follow-up learning activity? This would be of interest to readers.

Reviewer Expertise:

NA

No decision status is available

# Reviewer report: Reviewer response for version 1

**DOI:** 10.21956/mep.19709.r29486

**Published:** 2018-11-21

**Authors:** BALAJI ARUMUGAM

**Affiliations:** 1 TAGORE MEDICAL COLLEGE AND HOSPITAL

This review has been migrated. The reviewer awarded 4 stars out of 5 Congrats to the author for the good attempt to assess the student performance by self and tutors. The objectives were fulfilled by the clear administration of appropriate statistical tests. But as reviewer I would like to add or ask to the author regarding 1. Hybrid PBL was not explained in the study methods clearly 2. Performance in the four fundamental components of PBL: independent study, group interaction, active participation, and reasoning abilities - Was the objectives and the components were explained to the students those who participated in the study?3. Results of our study showed that the first year medical students consistently underscored their own performance in PBL tutorial sessions - may be also because of different or variety of TL methods were used during their block curriculum and less understanding of the PBL method and its objectives.4. the poor correlation between self-assessment and tutors assessment among first year compared with a strong correlation among second year students - may be due to number of PBL sessionsBut its a nice and well written manuscript on the newer TL methods and its assessment. Good work authors

Reviewer Expertise:

NA

No decision status is available
